# A Network Dynamics Model for the Transmission of COVID-19 in Diamond Princess and a Response to Reopen Large-Scale Public Facilities

**DOI:** 10.3390/healthcare10010139

**Published:** 2022-01-12

**Authors:** Yuchen Zhu, Ying Wang, Chunyu Li, Lili Liu, Chang Qi, Yan Jia, Kaili She, Tingxuan Liu, Huaiping Zhu, Xiujun Li

**Affiliations:** 1 Department of Biostatistics, School of Public Health, Cheeloo College of Medicine, Shandong University, Jinan 250012, China; zhuyuchenl@163.com (Y.Z.); lichunyu_biosta@163.com (C.L.); lili160629@163.com (L.L.); chanzyq@163.com (C.Q.); jiayan8hui@163.com (Y.J.); kailishe95@163.com (K.S.); liutx96@mail.sdu.edu.cn (T.L.); 2 Mathematical Institute, University of Oxford, Oxford OX2 6GG, UK; ying.wang-alice@outlook.com; 3 Mathematics and Statistics, York University, Toronto, ON M3J 1P3, Canada

**Keywords:** COVID-19, dynamics model, the Diamond Princess, network, public facilities

## Abstract

*Background*: The current epidemic of COVID-19 has become the new normal. However, the novel coronavirus is constantly mutating. In public transportation or large entertainment venues, it can spread more quickly once an infected person is introduced. This study aims to discuss whether large public facilities can be opened and operated under the current epidemic situation. *Methods*: The dual Barabási–Albert (DBA) model was used to build a contact network. A dynamics compartmental modeling framework was used to simulate the COVID-19 epidemic with different interventions on the Diamond Princess. *Results*: The effect of isolation only was minor. Regardless of the transmission rate of the virus, joint interventions can prevent 96.95% (95% CI: 96.70–97.15%) of infections. Compared with evacuating only passengers, evacuating the crew and passengers can avoid about 11.90% (95% CI: 11.83–12.06%) of infections; *Conclusions*: It is feasible to restore public transportation services and reopen large-scale public facilities if monitoring and testing can be in place. Evacuating all people as soon as possible is the most effective way to contain the outbreak in large-scale public facilities.

## 1. Introduction

COVID-19 suddenly broke out at the end of 2019. At that time, people lacked sufficient awareness of the novel coronavirus and could not take timely measures to deal with the spread. The virus spread through droplets and contact [1] and easily spread in densely populated places, especially entertainment venues and public facilities (such as cruise ships, airplanes). In these places, the poor air ventilation and strong personnel mobility can cause widespread transmission once an infected person appears. For example, more than a dozen large cruise ships at that time were all affected by COVID-19, especially the Diamond Princess [2].

Currently, measures to combat COVID-19 are becoming more efficient. Although the epidemic is still spreading, the prevention and control efforts in various countries have entered the stage of regular management. For example, China’s normalized management of COVID-19 corresponds to a “containment goal”, which means the complete interruption of local transmission of COVID-19 [3]. Up to now, the current epidemic of COVID-19 has become a normalized situation, whereby most countries have been able to keep the number of cases to a low level. However, because of virus mutation [4,5], there are still local outbreaks in some countries and regions [6]. Especially in large-scale public facilities such as transportation and entertainment venues that are essential for public normal life, local outbreaks are more likely to occur. In the early stages of the epidemic, most countries chose to ban the gathering of large groups, close large supermarkets and schools, and reduce the number of flights and cruises to avoid the spread of the virus [7,8]. However, as schools, factories, and the like gradually open up, these strict prevention and control measures have become a challenge for public health. Therefore, whether public facilities can be fully opened as usual, and whether transmission can be controlled in a timely and effective manner if an epidemic occurs are important issues that needs to be addressed.

In order to answer the above questions, we take the Diamond Princess cruise ship as a case to build an infectious disease network dynamics model for illustration. Due to insufficient awareness of COVID-19 at the time of the outbreak, effective prevention and control measures were not taken on the cruise ships, resulting in large-scale spread. Therefore, on the basis of the actual situation of the ship, we simulated the COVID-19 epidemic when different control measures were implemented. The implementation of the control measures can be assessed through changes in the total number of infections to explore the possibility of opening up public places and transportation facilities.

The population structure, age stratification, population activities, and community structure between different regions will all have an impact on the dynamics of the epidemic. For example, people on the same train have a greater chance of contacting people in the same coach than people in different coaches. Studies have also shown that the structure of the contact network is essential to explain the mode of transmission of infectious diseases, especially for directly transmitted diseases [9,10,11,12]. Valdez et al. built a model with a community network to estimate the probability of a pandemic [13]. Liu et al. used a contact network to simulate the epidemic of the Diamond Princess and obtained $R_{0}$, but the SIR (Susceptible, Infected, Recovered) network model they used may have ignored the impact of the incubation period and overestimated the early transmission capacity of COVID-19 [14]. In summary, constructing a network model with a community structure will allow us to better understand of the transmission process. In order to increase the versatility of the model, we also modified the transmission rate of COVID-19 and the ratio of asymptomatic infections to simulate how to control other similar respiratory infectious diseases.

For large-scale public facilities such as cruise ships, trains, and airplanes, people’s behaviors are significantly more active than in daily life, and close contact can easily cause the spread of infectious diseases. In order to evaluate whether large-scale public facilities can be reopened normally, our study simulated COVID-19 in different scenarios and estimated the impact of interventions on the spread of COVID-19. In the context of the normalization of the epidemic, our research can provide a reference for the prevention and control of new types of infectious diseases or other public health emergencies.

## 2. Materials and Methods

### 2.1. Data Sources

The daily reported data of COVID-19 cases in this study were from the Ministry of Health, Labor, and Welfare of Japan. The age distribution of all members of the Diamond Princess was reported by the National Institute of Infectious Diseases (NIID) in Japan [15].

### 2.2. Epidemic Simulation Model

We assumed that no vaccine was available during the period of the endemic of Diamond Princess. Vaccination reduces the chance of the virus spreading; hence, the situation after vaccination would be improved. In addition, because of novel coronavirus mutations, some vaccinated people can still be infected with the novel coronavirus. Therefore, the assumption that no one is vaccinated may have more practical significance for future epidemic prevention and control.

**Figure 1:**
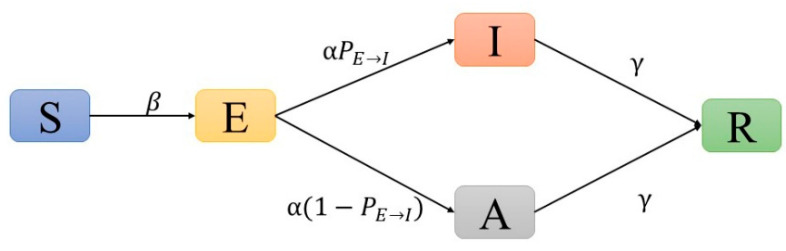
The structure of SEIAR model.

We used the following settings: individuals were represented as nodes in the network, and edges represented contact between connected nodes. Each node was in one of five possible states: (S) susceptible, (E) exposed, (I) infected (with symptomatic), (A) infected (asymptomatic), or (R) removed (recovered and death) (see Supplementary Materials). At each time step (1 day), an S node was converted to an E node with a probability (β), which could be converted to an I node with a fixed probability $P_{E\to I}$ after the incubation period (1/α) or converted to an A node with the probability of $1-P_{E\to I}$. The infectivity of asymptomatic cases (A) was less than that of infected cases (I). According to the study of Hou et al. [16], we set this proportion to 40%. Both A and I nodes were converted to R nodes after going through the recovery period (1/γ) (Figure 1, Table S1).

In order to mimic the epidemic process, we set a serial interval (SI) that follows the Gamma distribution (Gamma 6, 1.2) for each individual [1,17,18]. The setting of all the above parameters were consistent with COVID-19, which was used as the baseline (Table S1). Moreover, we conducted a sensitivity analysis on the proportion of asymptomatic infections to explore the impact of asymptomatic infections on the outbreak of COVID-19. For virus mutations and similar respiratory infectious diseases, we modified the value of β (−50%, +50%, +100%) to observe whether interventions had different effects on different mutated strains.

### 2.3. Epidemic Simulation Model Contact Network Generation

In large public facilities, the contact network among people is not a fully connected network of equal weight. For example, the probability of contact between staff (crew) and consumers (passengers) is different from the probability of contact between consumers and consumers. In order to better simulate the contact network among people, we adopted the dual Barabási–Albert (DBA) model [19] to simulate the contact network structure of the Diamond Princess. The DBA model can better capture these attributes of real social contact networks. The parameter settings resulted in the average network degree of each simulation being between 40 and 50, which was slightly higher than the approximate number of contacts in the first case reported on the day [14]. In each simulation, 8–15 communities could be obtained in the network, and the connections among nodes within the community were closer than the connections among the communities (see Supplementary Materials).

Since we could only obtain the distribution of ages, we randomly generated ages from each age group with a uniform distribution. For example, if a node was in the 20–29 age group, we used uniform (20,29) as the age of this node. Furthermore, we assumed a positive linear correlation between the age of each individual and the probability of being infected (β). Since the number of people under 10 years old was very small (<0.5%), the problem of low resistance of children was ignored.

Moreover, people’s social behaviors can cause changes in the network structure, which will have an impact on the spread of diseases. Therefore, we set the parameter $p_{structure\;}$ (default = 0) to represent the probability of disconnecting an old connection and the probability of generating a new connection. A larger $p_{structure\;}$ denotes greater instability of the network structure.

### 2.4. Management of Customers

The management of consumers is reflected in two approaches (isolation and evacuation). One approach is to reduce the possibility of contact, which specifically includes measures such as isolation and wearing masks (only “isolation” on the Diamond Princess). The other is to disconnect all nodes in the network, including evacuating public facilities directly or evacuating after “PCR” testing. In order to evaluate whether these management measures can effectively control the spread of the disease after infected persons appear, we set up the scenarios described below for simulation.

The scenario without any interventions was considered as the baseline. We assumed that, for a new type of infectious disease (such as COVID-19), the time to implement intervention measures was determined by the number of cases (I) that can be observed on the cruise ship. The condition for two management measures to be implemented was when the number of cases reached a certain number, and we recorded this value as $n_{start}$. Isolation was divided into two cases according to the possibility of disconnection ($p_{isolation}$ = 0.9, 0.9999) of each edge in the network. We assumed that evacuations last only 7 days, and the number of people evacuated every day was the same because of the high cost. The number of people evacuated every day was divided into high and low levels ($n_{evacuate}$ = 380, 50). Except for those who were infectious (I), people in other states had the same probability of being selected for evacuation. In addition, we also compared the scenario where only the passengers were evacuated (the actual situation) and both crew and passengers were evacuated.

Since these two interventions can be carried out at the same time, we also simulated the effect of joint interventions. The 22 detailed scenario settings are shown in Table 1 and Table S2. The infection of nodes in the network was stochastic; hence, the simulation in each scenario needed to be executed at least 50 times to reduce the deviation caused by stochasticity. If a certain simulation did not cause an outbreak, it was considered an outlier, and the result was removed. The epidemic curves of COVID-19 could be obtained by taking the average of the 50 simulations. At the same time, we could also use the bootstrap method to obtain the 95% CI. The duration of each simulation was 80 days, but the calculation of the total number of cases was as of the 50th day (when the quarantine on the Diamond Princess was completed). In addition, we also recorded the ages of all infected persons to compare the differences in age distribution between simulated and real data. All simulations were conducted using Python3.8.

## 3. Results

**Figure 2:**
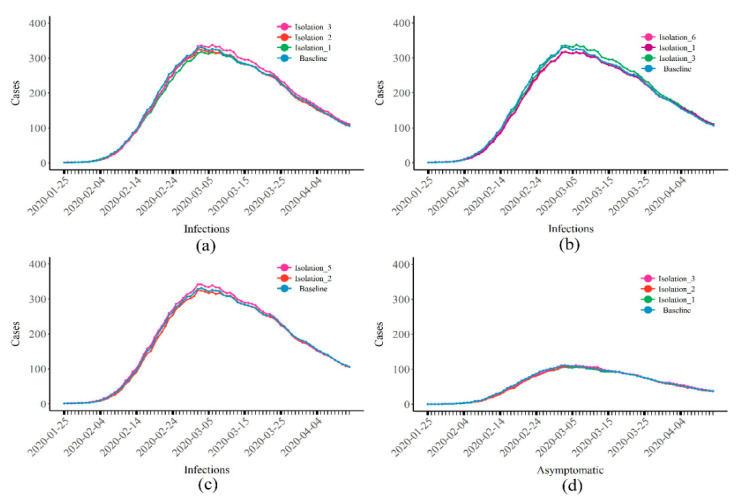
The epidemic curves of COVID-19 when the control measure was isolation only. (**a**,**b**) The change in the number of infections according to (**a**) the start time or (**b**) the intensity of isolation; (**c**) the number of infections when the social intention was changed; (**d**) the number of asymptomatic infections. The number of cases represents the people in state I on that day, not the number of newly reported cases each day.

Figure 2 and Table 2 show a comparison with the baseline situation where only isolation was in place. A lower number of cases when isolation started led to a lower total number of cases. Whether $n_{start}$ (the number of infections when isolation started) reached 10, 20, or 50, the total number of cases that could be reduced by isolation was limited (Figure 2a). If isolation started at $n_{start}$ = 10 (Isolation 1), a total of 1380 people (95% CI: 1256–1488) were infected. Compared with the baseline (1445, 95% CI: 1334–1546), only 65 cases were prevented. When the strength of isolation was increased ($p_{isolation}$ = 0.999), the total number of cases was reduced to 1353 (95% CI: 1254–1452). Compared with Isolation 1, only 27 cases were prevented (Figure 2b). If the social intention ($p_{structure\;}$) was increased (Isolations 1 and 5), the total number of cases exceeded the baseline, reaching 1497 (95% CI: 1418–1560, Figure 2c). The epidemic curves of asymptomatic infections exhibited similar results (Figure 2d).

**Figure 3:**
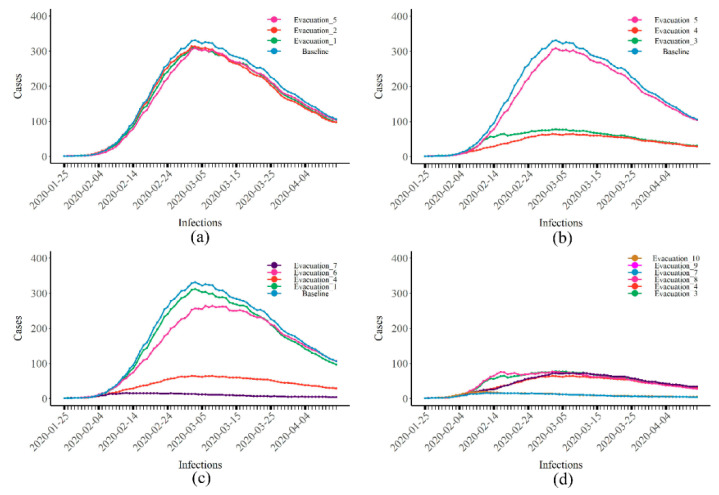
The epidemic curves of COVID-19 when the control measure was evacuation. (**a**) Results of changing the start time of evacuation when the number of evacuees was low; (**b**,**c**) results of changing (**b**) the number of evacuees and (**c**) the identity of evacuees; (**d**) results of changing the social intentions of passengers when the number of evacuees was high. The number of cases represents people in state I on that day, not the number of newly reported cases each day.

When only evacuations were applied, if the number of people that could be evacuated every day was limited ($n_{evacuate}$ = 50), earlier evacuations led to a lower total number of cases (Figure 3a). Late evacuations resulted in a large number of people that could be evacuated every day ($n_{evacuate}$ = 380, $n_{start}$ = 50), with a total number of cases of 335 (95% CI: 309–361), i.e., a 76.82% reduction compared with the baseline (Figure 3b). Under the same conditions, if only passengers were evacuated, the effect was reduced. For example, when $n_{evacuate}$ = 50 and $n_{start}$ = 10, evacuating both passengers and crew (pink) reduced the total number of cases to 1109 (95% CI: 1027–1176). This was a decrease of 11.04% compared to when only passengers were evacuated (green) (Figure 3c). In addition, when the social intentions of people increased, the total number of cases reduced by evacuations also decreased (Figure 3d), similar to the results of isolation.

**Figure 4:**
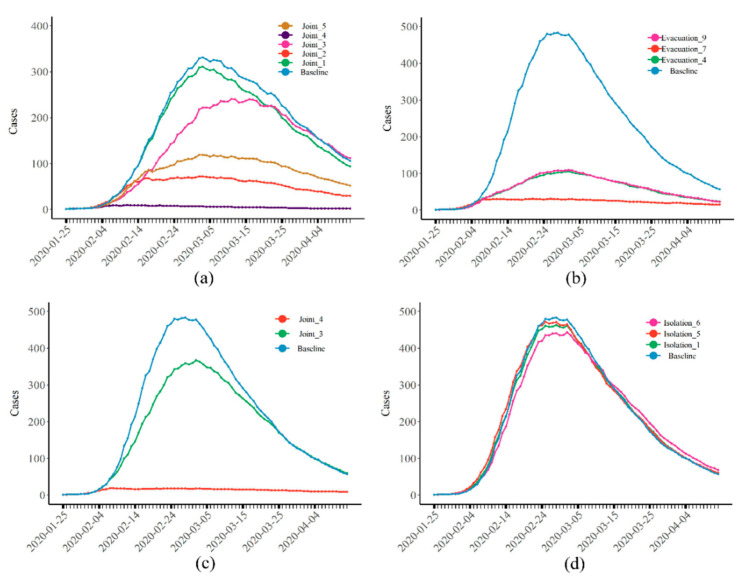
(**a**) The epidemic curves of COVID-19 when implementing joint interventions; (**b**–**d**) prevalence of infectious diseases after (**b**) isolation, (**c**) evacuation, and (**d**) joint intervention measures when the transmission rate increased by 50%. The number of all cases represents the sum of people infected that day, not the number of new cases.

Compared with separately implementing these two interventions, the joint interventions had the best effect. When $n_{start}$ was greater than 5 and both interventions were implemented, the total number of cases was reduced to 44 (95% CI: 38–51), i.e., a 96.95% reduction compared with the baseline. If the number of evacuees everyday was low ($n_{evacuate}$ = 50), and the interventions were implemented earlier (Joint 3, pink), the total number of cases was reduced by 34.94% (Figure 4a). Figure 4b–d show the epidemic curves of infectious disease when the transmission rate was increased by 50% compared to COVID-19. It can be seen that both types of interventions could reduce the peak number of daily infections and the total number of cases, while joint interventions were still the best. Moreover, we found that, despite the increased infectivity of the infectious disease, strong interventions could still effectively maintain the total number of cases (108, 95% CI: 85–137) at a low level. According to the above results, we estimate that, under real circumstances, the isolation intervention implemented on the Diamond Princess was almost ineffective, whereas the evacuation intervention played a decisive role. However, due to the small number of evacuees and the late evacuation, the epidemic of COVID-19 was not controlled.

**Figure 5:**
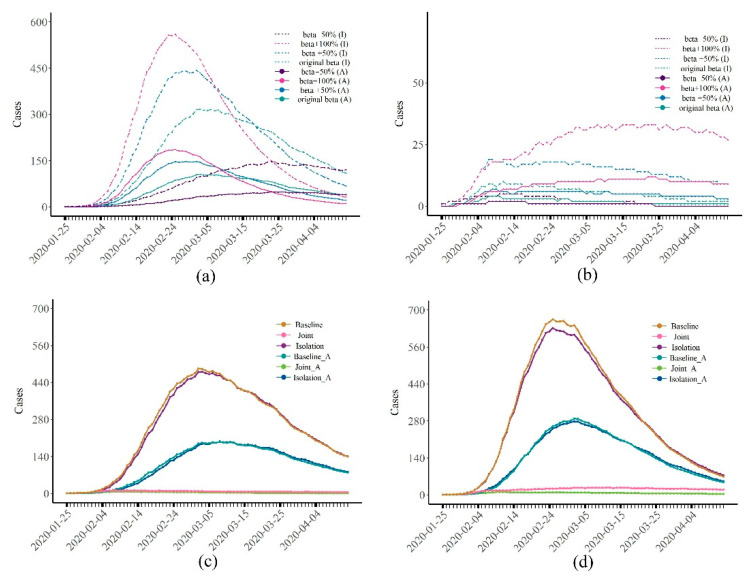
(**a**,**b**) Results of the (**a**) isolation and (**b**) joint interventions under four transmission rates; (**c**) transmission rate of COVID-19 and (**d**) sensitivity analysis to asymptomatic infections when the transmission rate increased by 50%; (**c**) results of COVID-19 and (**d**) theoretical infectious disease when the transmission rate increased by 50%. Baseline, Isolation, and Joint denote an epidemic of infectious diseases when there were no asymptomatic infections. Baseline_A, Isolation_A, and Joint_A denote an epidemic of infectious diseases when the proportion of asymptomatic infections increased to 50%. Isolation refers to Isolation 6 in Table 1 and Joint refers to Joint 4 in Table 1.

Figure 5a,b show the epidemic curves of the disease when the infectivity of the virus (β) increased by 50% (or 100%) and decreased by 50%. Consistent with our previous findings, despite changes in the infectious power of the disease, the number of cases that could be reduced by isolation was still very limited, with an average reduction in the total number of cases of about 4% (Figure 5a, Table S2). Joint interventions could effectively control the spread of disease. When the transmission rate (β) increased by 100%, 2749 (95% CI: 2671–2814) people were infected without any intervention. Under the strongest joint intervention approach (Joint 4), the total number of cases was reduced to 167 (95% CI: 147–187).

The results of the sensitivity analysis of the proportion of asymptomatic infections are shown in Figure 5c,d. It can be found that the effect of isolation only was still not good, while joint interventions could still effectively control the epidemic. When there were no asymptomatic infections, the slope of the epidemic curve slowed down, the arrival of the peak was delayed, and the total number of cases decreased accordingly. These findings suggest that the presence of asymptomatic infections (A) increases the chance of susceptible people being infected; when there are no asymptomatic infections, the infectious disease can be better controlled.

In addition, the age distribution of all cases and the COVID-19 epidemic curves of passengers and crew are shown in Supplementary Figures S1 and S2, respectively. When isolation and joint interventions were implemented, the age distributions of cases were the same as the reported cases on the Diamond Princess. When only isolation was implemented, the peaks of the epidemic curves for crew and passengers appeared on the same day (Figure S2a). However, if the evacuation intervention was implemented, the peak of passengers arrived first, and the peak of the crew arrived 14 days later (Figure S2b). The same result was obtained for the joint interventions (Figure S2c).

## 4. Discussion

In this study, taking the Diamond Princess cruise ship as an example, we built a network model for the passengers and crew to obtain the impact of social contact on the spread of the epidemic. We revised the implementation time and implementation intensity of different control measures to evaluate their validity. Our model is also suitable for the situation of virus mutation, which can provide new insights for combating COVID-19. Therefore, we can respond to the question whether large-scale public facilities can be reopened in the context of COVID-19 normalization.

Different control measures have different effects in responding to sudden epidemics, but rapid control measures can effectively control the epidemic. The effect of isolation itself was rather limited regardless of the transmission rate. The reason may be that when the outbreak first occurred, activities on the Diamond Princess proceeded normally. People who were in the incubation period or became asymptomatic were not identified. Despite the isolation measures taken, the crew still needs to provide meals and other services to passengers. Therefore, there are still connections between nodes, and the virus can continue to spread. If the connections (edges) in the network cannot be completely cut off, as long as there is an edge between two nodes, people will have the chance to be infected, and the effect of complete isolation will not be realized. Thus, the outbreak is almost inevitable. In addition, the staff who boarded the ship for quarantine found that the quarantine area was not clearly demarcated, and the disinfection equipment was disorderly arranged. These signs all indicate that the virus may have still continued to spread through human-to-human contact [20,21].

Evacuation is different from isolation because the evacuated nodes are removed from the entire network. This means that, if there are asymptomatic infections or people in the incubation period among the evacuees, they will no longer have the opportunity to spread the virus. This may be one of the reasons why evacuation is more effective. In addition, when only evacuating passengers, fewer people can avoid being infected than when evacuating both passengers and crew. This may be because the crew needs to work all the time; hence, they have a larger degree in the network than passengers.

The effect of joint intervention was best but also the most difficult to implement. For the Diamond Princess, passengers could return home country only when their government dispatch chartered flights. However, for cruise ships (Table S3) in a similar situation to the Diamond Princess, their managers disembarked all people and applied “PCR testing” (COVID-19 polymerase chain reaction test) to avoid more cases. Corresponding to the current normalization of the COVID-19 epidemic, promptly organizing personnel to evacuate public facilities and applying “PCR testing” for all evacuees is an effective means to curb the spread of the virus.

By changing the transmission rate of the virus and the proportion of asymptomatic infections, we also simulated the scenarios of virus mutation (or other respiratory diseases). When the transmission rate increased, the total number of infections increased. In addition, the increase in the proportion of asymptomatic infections also led to more cases, because most asymptomatic infections are difficult to detect in the early stages of the epidemic without a test. However, the simulation results showed that rapid and effective management measures can quickly control the epidemic. This shows that the current control measures are very necessary. If measures cannot be taken quickly, thus will not only increase the duration of the epidemic, but also lead to more cases, resulting in an uncontrollable situation.

Our simulation results are not completely consistent with the number of reported cases on the Diamond Princess cruise ship. There are several possible reasons. First, it was unrealistic to allow all infected persons to be tested when COVID-19 first began to spread [22]. Second, when taking a “PCR test” on the Diamond Princess, priority was given to passengers, especially those who were older and had complications, while the crew was considered last. If there were not enough reagents to test all people in time, the crew could not take a “PCR test” [23]. Because the number of reported cases was not in line with the actual number of cases, incorrect parameter estimates could occur. Therefore, we did not estimate the parameters on the basis of the actual number of cases; instead, we used the parameters reported in other studies instead of fitting the data to obtain the parameters [16,17,24,25,26,27] (Table S1).

Our study also explained some phenomena of the COVID-19 epidemic aboard the Diamond Princess. We found that the reasons for the peak time of the crew being later than the peak time of the passengers were not only due to the “detection sequence”, but also related to the evacuation measures. If no evacuation measures were implemented, the two peaks would have occurred at the same time. In addition, the age distribution of cases was related to isolation measures. If isolation measures (including joint interventions) were not implemented, the age distribution of cases would have been inconsistent with reality. This shows that isolation measures were indeed taken on cruise ships, but only to a limited effect.

In general, the rapid implementation of effective interventions can prevent the outbreak of infectious diseases in large public facilities. The current epidemic of COVID-19 has become a normalized situation. Although the virus is still mutating, the various prevention and control measures have become more mature. Once a suspected case is found, both the cases and the people in close contact are “PCR tested”, and the facility is shut down quickly (for example, the cruise ship would immediately organize all people to disembark). This is the same as the evacuation measures on cruise ships, which directly sever connections with other people in the network, thereby effectively preventing the outbreak of COVID-19. In summary, it is feasible to restore public transportation and open up large public facilities, such as the restoration of flights and cruise voyages.

## 5. Conclusions

In the context of COVID-19 normalization, it is feasible to restore public transportation services and reopen large-scale public facilities provided monitoring and testing are in place. Regardless of the transmission rate of the virus, evacuating all people as soon as possible is the most effective way to contain the outbreak. Compared with evacuation, the implementation of isolation measures is more difficult, and it is harder to achieve the desired effects. In addition, higher social intentions of consumers and the proportion of asymptomatic infections increase the difficulty of controlling the epidemic. Rapid and timely monitoring and testing can effectively contain COVID-19.

## Figures and Tables

**Table 1:** Setting of 22 intervention scenarios.

**Interventions**	**Number of Infections When Isolation Started** ** $\;(n_{start})$ **	**Proportion of Edges Removed** $\;(p_{isolation})$	**Network Stability** $\;(p_{stability})$	**Number of Infections When Evacuation Started** $\;(n_{start})\;$	**Number of Evacuees (/day)** $\;(n_{evacuate})$	Identity of Evacuees
Baseline						
Isolation 1	>10	0.9				
Isolation 2	>20	0.9				
Isolation 3	>50	0.9				
Isolation 4	>50	0.9999				
Isolation 5	>10	0.9	0.2			
Isolation 6	>10	0.9999				
Baseline						
Evacuation 0				Actual *		
Evacuation 1				>10	50	Passenger
Evacuation 2				>20	50	Passenger
Evacuation 3				>50	380	Passenger
Evacuation 4				>10	380	Passenger
Evacuation 5				>50	50	Passenger
Evacuation 6				>10	50	Total
Evacuation 7				>10	380	Total
Evacuation 8			0.2	>50	380	Passenger
Evacuation 9			0.2	>10	380	Passenger
Evacuation 10			0.2	>10	380	Total
Joint 1	>10	0.9		>10	50	Passenger
Joint 2	>50	0.9		>50	380	Passenger
Joint 3	>10	0.9999		>10	50	Total
Joint 4	>5	0.9999		>5	380	Total
Joint 5	>50	0.9	0.2	>50	380	Passenger

Note: * indicates the real situation of evacuations. From 17 February to 23 February, each country evacuated its own nationals in batches by air. The details are shown in Supplementary Figure S3. “Number of cases when isolation (evacuation) started” means that when the number of people in the I state reached a certain value (10, 20, 50), the isolation (evacuation) intervention was implemented.

**Table 2:** The prevalence of infectious diseases in different scenarios.

Interventions	Peak Time	Peak Value	95% CI of Peak	Total Cases	95% CI of Cases
Baseline	03-03	331	307–349	1445	1334–1546
Isolation 1	03-03	318	296–336	1380	1256–1488
Isolation 2	03-02	325	301–343	1394	1283–1494
Isolation 3	03-06	338	332–345	1428	1354–1494
Isolation 4	03-06	325	311–337	1394	1296–1500
Isolation 5	03-02	342	329–353	1497	1418–1560
Isolation 6	03-03	317	294–337	1353	1254–1452
Baseline	03-03	215	201–227	906	827–979
Evacuation 0	03-03	312	293–326	1332	1243–1414
Evacuation 1	03-02	314	295–328	1388	1299–1471
Evacuation 2	03-03	78	74–82	335	309–361
Evacuation 3	03-08	65	58–71	301	268–331
Evacuation 4	03-03	309	288–325	1273	1173–1363
Evacuation 5	03-08	264	256–272	1109	1027–1176
Evacuation 6	02-12	16	13–18	74	65–84
Evacuation 7	03-02	77	72–81	356	336–377
Evacuation 8	03-08	73	69–77	323	299–345
Evacuation 9	02-11	19	16–23	84	73–95
Evacuation 10	03-03	311	299–320	1364	1287–1428
Joint 1	03-02	72	67–77	341	308–369
Joint 2	03-09	241	228–250	940	866–1022
Joint 3	02-11	10	8–12	44	38–51
Joint 4	03-02	119	111–126	528	487–571
Joint 5	03-03	331	307–349	1445	1334–1546

Note: The peak number of cases in the table are all status values, i.e., the number of people who were in state I in that day, not the number of newly reported cases each day.

## Data Availability

The daily reported data of COVID-19 cases in this study were from the Ministry of Health, Labor, and Welfare of Japan. The age distribution of all members of the Diamond Princess were reported by the National Institute of Infectious Diseases (NIID) in Japan (https://www.niid.go.jp/niid/en/2019-ncov-e/9407-covid-dp-fe-01.html, accessed on 18 December 2020).

## Supplementary Materials

The following are available online at https://www.mdpi.com/article/10.3390/healthcare10010139/s1: Text S1. Model supplement; Figure S1. Box plot of the ages of all cases on the Diamond Princess; Figure S2. COVID-19 epidemic curves of people with different identities (passengers, crew, total) under different interventions; Figure S3. Timeline of the outbreak on the Diamond Princess; Table S1. Parameters of COVID-19; Table S2. Epidemic curves of infectious diseases when changing transmission rate (β); Table S3. Quarantine and port information of three cruises.
