# Supplementary material for: A Network Dynamics Model for the Transmission of COVID-19 in Diamond Princess and a Response to Reopen Large-Scale Public Facilities
Source: Healthcare (Basel). 2022 Jan 12;10(1):139. doi: 10.3390/healthcare10010139 (PMC8775839; doi:10.3390/healthcare10010139)
Supplement: Supplementary file 1 [file healthcare-10-00139-s001.zip › healthcare-1510467-supplementary.pdf]

Model supplement:

Network model

The structure of the SEIAR dynamic model adopted is as follows:

$$\frac{dS}{dt} = -\beta S(t)$$

$$\frac{dE}{dt} = \beta S(t) - \alpha P_{E\to I} E(t) - (1-\alpha) P_{E\to A} E(t)$$

$$\frac{dI}{dt} = \alpha P_{E\to I} E(t) - \gamma I(t)$$

$$\frac{dA}{dt} = (1-\alpha) P_{E\to A} E(t) - \gamma A(t)$$

$$\frac{dR}{dt} = \gamma (I(t) + A(t))$$

$$S(t) + E(t) + I(t) + A(t) + R(t) = 1.$$

In the network, each node was in one of the following 5 possible states: (S) susceptible, (E) exposed, (I) infected (with symptomatic), (A) infected (asymptomatic) or (R) removed (recovered and death). The contact network has no direction, no weight, and no self-loop (that is, all contacts are mutual, and all contacts spread infection with the same probability). Initially, all nodes are S. Then one susceptible node will be randomly selected as I, and an integer between 1-5 will be randomly selected as the number of E to complete the initial state setting.

In order to simulate the closeness of connections between different types of people in the contact network, the complete complex network is generated by the DBA preferential attachment network model. A network of n nodes is grown by attaching new nodes each with either m_1 edges (with probability p) or m_2 edges (with probability 1−p) that are preferentially attached to existing nodes with high degree. In this study, we set it up as follows. The parameters, $m_1 = 0.03 \times \mathit{the\ number\ of\ paasengers}$, represents the average number of individuals that a passenger can contact, $m_2 = 0.03 \times \mathit{the\ number\ of\ crews}$, represents the average number of individuals that a crew can contact, p is approximately the proportion of

passengers in the total number of people, and n=3711.

Interventions

In order to judge whether the implementation of isolation is effective, we set the following scenarios: (1) When the number of infections (I) (n_{start})>10, 20, 50, the quarantine will start. (2) After (1) is satisfied, set the probability of each edge being cut off ($p_{isolation}$) to 0.9 or 0.9999， where 0.9999 means that more stringent isolation has been implemented. (3) When the heterogeneity of the population h=0, 0.2, press (1) and (2) to intervene. Another type of intervention is the evacuations, which are determined by the particularity of the cruise ship. Because the ship is not allowed to dock, countries can only send charter flights to pick up passengers.

Due to the high cost of evacuations, we assume that evacuations last only 7 days, and the number of people evacuated every day is the same. Except for those who are infections (I), the rest have the same probability of being selected for evacuation. We set the following scenarios to determine the validity of the evacuations: When the number of infections (I) (n_{start})>10, 20, 50, the evacuations will start. (2) After (1) is satisfied, the number of evacuees ($n_{evacuate}$) per day =50 or 380.And 380 indicates that the intensity of evacuation is higher. (3) Whether to increase heterogeneity on the basis of (1) and (2), i.e. h =0 or 0.2. (4) Evacuation of passengers only (actual situation) or both passengers and crew can be selected to evacuate. In addition, we also need to check the effect of joint intervention (simultaneous isolation and evacuation). The detailed scenario settings are shown in Table 1.

Figures

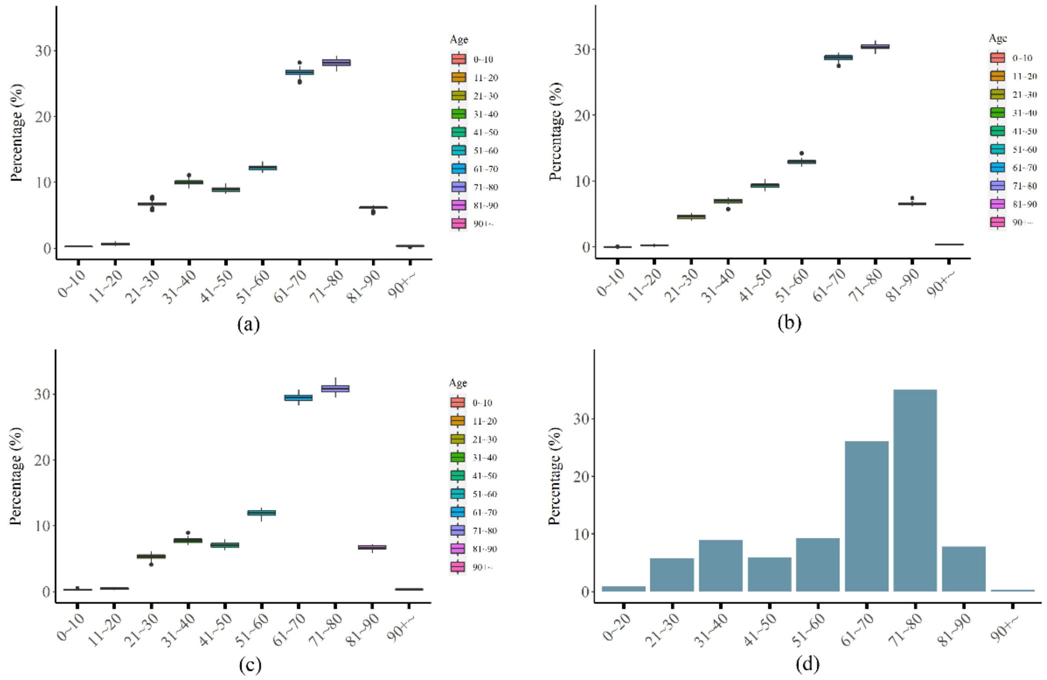

Figure S1. Box plot of the ages of all cases on the Diamond Princess. (a), (b), (c) respectively represented the age distribution of all infections when the control measures were Isolation 2, Evacuation 2, And Joint 3; (d) represented the age distribution of the Diamond Princess.

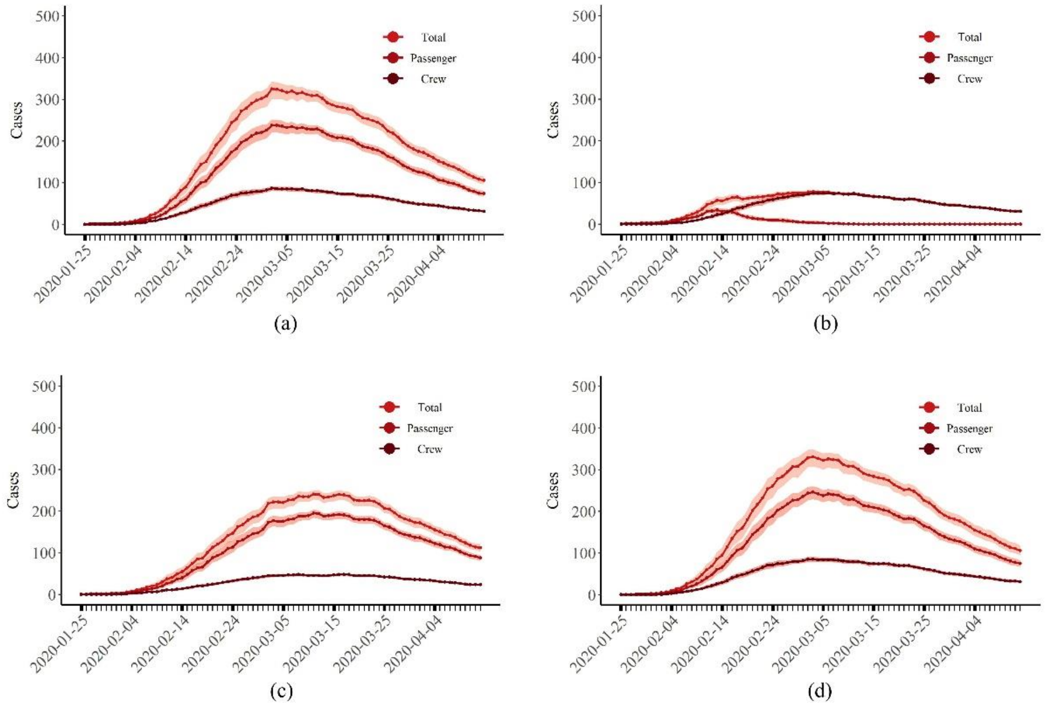

Figure S2. The COVID-19 epidemic curves of people with different identities

(passengers, crew, total) under different interventions. (a), (b), (c) represented the intervention scenarios of Isolation 2, Evacuation 3 and Joint 3 respectively; (d) indicated the results of Baseline.

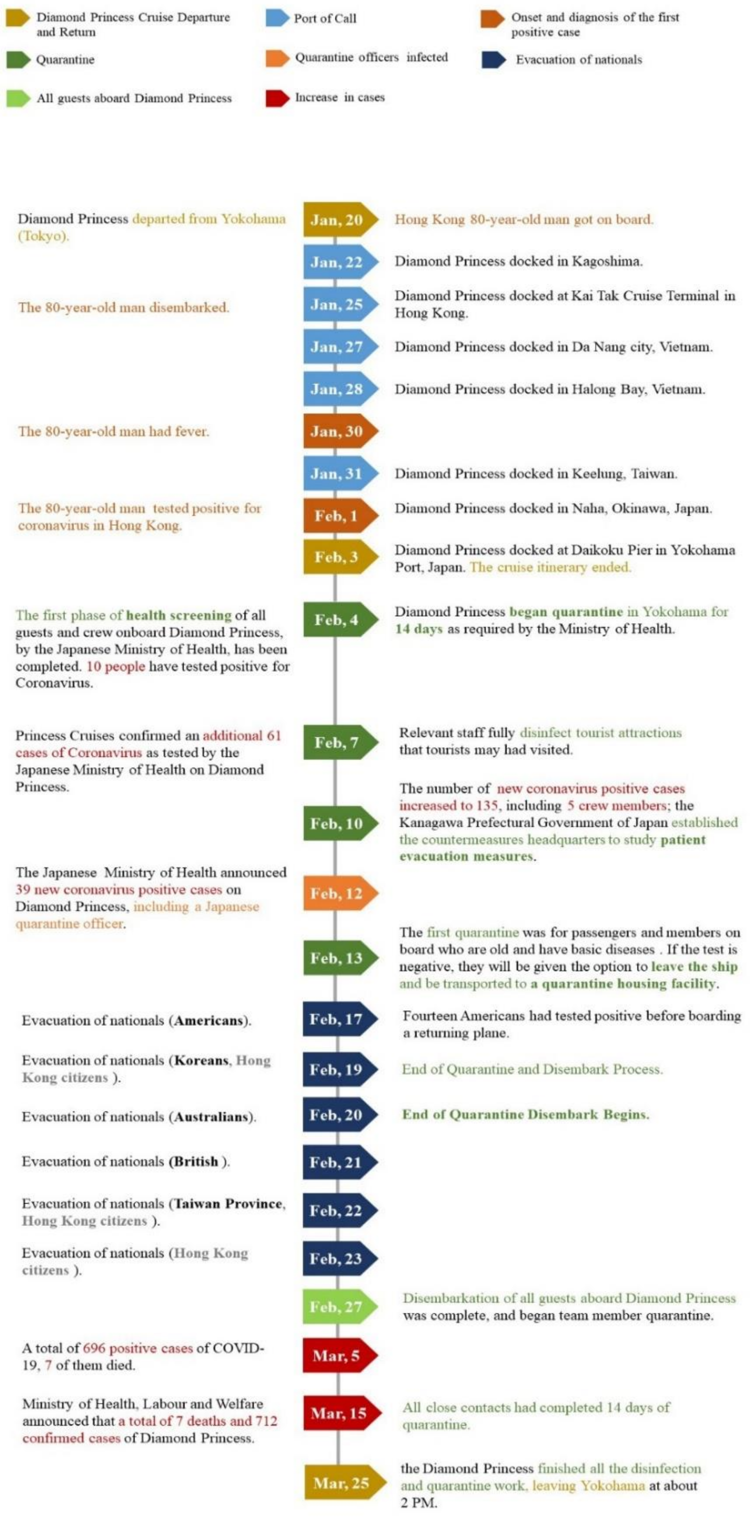

Figure S3. The timeline of the outbreak on the Diamond Princess. The arrow icon indicates the time of the event, and different colors represent different types of events.

Tables

Table S1. The parameters of COVID-19

Parameter	Value	References
R_0	2.5	[1] *
Incubation period($1/\alpha$)	4	[2–4]
Recovery period($1/\gamma$)	7	[3, 4]
Serial interval	Gamma(6,1.2)	[5–7]
Probability of E to A (1-$P_{E\to I}$)	30%	[8–12]
The transmission rate ratio of A to I	0.4	[12, 13]

Note: * indicates that the estimated value of the basic reproduction number (R_0) is obtained from Reference 1 and the estimated value in this study.

Table S2. The epidemic curves of infectious diseases when Transmission rate(β) are changed

Interventions	Peak time	Peak value	Peak 95%CI	Total cases	Cases 95%CI
β (+50%)					
Baseline	02-28	483	472-492	2270	2194-2339
Isolation 1	03-02	106	103-110	513	484-536
Isolation 5	02-21	31	26-35	181	164-201
Isolation 6	03-02	109	107-112	525	502-544
Evacuation 4	02-28	463	447-475	2220	2142-2296
Evacuation 7	02-25	472	447-490	2273	2173-2351
Evacuation 9	03-02	443	409-467	2116	1967-2235
Joint 3	03-02	367	342-388	1704	1561-1811
Joint 4	02-07	19	13-26	108	85-137
β (+100%)					
Baseline	02-23	590	559-610	2749	2671-2814
Isolation 6	02-25	560	530-587	2632	2502-2736
Joint 4	03-16	33	30-37	167	147-187
β (-50%)					
Baseline	03-23	156	139-168	412	352-478
Isolation 6	03-23	147	136-157	417	364-474
Joint 4	02-13	5	4-7	17	15-19

Table S3. Quarantine and port information of 3 cruises

Cruise name	Confirmed Cases	Days before moored	Quarantine start time	Passenger disembarkation time	Quarantine method	Measures

Diamond Princess	712 (A total of 3618 people were tested and 11 died)	15	2020-02-03	2020-02-03	Quarantine personnel on board quarantine	A national warning was issued on January 31: Passengers are required to isolate themselves. Quarantine began on February 4. On February 7, the sightseeing spots were fully disinfected; on February 12, passenger voluntary disembarkation plans were carried out in stages.
Grand Princess	103 (A total of 1103 people were tested and 3 died)	27	2020-03-09	2020-03-09	Quarantine personnel on board quarantine	On March 5, after the ship docked, the rescue team went to the Supreme Princess Airdrop Kit to detect coronavirus on passengers on the ship.
Costa Serena	0	4	2020-01-23	2020-01-24	docked and then quarantined	On January 25, all passengers disembarked, except 15 passengers with fever and negative virus test.

References

1. Wu JT, Leung K, Leung GM. Nowcasting and forecasting the potential domestic and international spread of the 2019-nCoV outbreak originating in Wuhan, China: a modelling study. Lancet. 2020;395:689–97. doi:10.1016/S0140-6736(20)30260-9.

2. Lauer SA, Grantz KH, Bi Q, Jones FK, Zheng Q, Meredith HR, et al. The Incubation Period of Coronavirus Disease 2019 (COVID-19) From Publicly Reported Confirmed Cases: Estimation and Application. Ann Intern Med. 2020;172:577–82.

3. Li R, Pei S, Chen B, Song Y, Zhang T, Yang W, et al. Substantial undocumented infection facilitates the rapid dissemination of novel coronavirus (SARS-CoV-2). Science. 2020;368:489–93.

4. Song R, Han B, Song M, Wang L, Conlon CP, Dong T, et al. Clinical and epidemiological features of COVID-19 family clusters in Beijing, China. J Infect. 2020;81:e26–30.

5. Li Q, Guan X, Wu P, Wang X, Zhou L, Tong Y, et al. Early Transmission Dynamics in Wuhan, China, of Novel Coronavirus-Infected Pneumonia. N Engl J Med. 2020;382:1199–207.

6. Zhang J, Litvinova M, Wang W, Wang Y, Deng X, Chen X, et al. Evolving epidemiology and transmission dynamics of coronavirus disease 2019 outside Hubei province, China: a descriptive and modelling study. Lancet Infect Dis. 2020;20:793–802. doi:10.1016/S1473-3099(20)30230-9.

7. Bi Q, Wu Y, Mei S, Ye C, Zou X, Zhang Z, et al. Epidemiology and transmission of COVID-19 in 391 cases and 1286 of their close contacts in Shenzhen, China: a retrospective cohort study. Lancet Infect Dis. 2020;20:911–9. doi:10.1016/S1473-3099(20)30287-5.

8. Li R, Pei S, Chen B, Song Y, Zhang T, Yang W, et al. Substantial undocumented infection facilitates the rapid dissemination of novel coronavirus (SARS-CoV-2). Science. 2020;368:489–93. doi:10.1126/science.abb3221.

9. Mizumoto K, Kagaya K, Zarebski A, Chowell G. Estimating the asymptomatic proportion of coronavirus disease 2019 (COVID-19) cases on board the Diamond Princess cruise ship, Yokohama, Japan, 2020. Euro Surveill. 2020;25.

10. Zhao S, Musa SS, Lin Q, Ran J, Yang G, Wang W, et al. Estimating the Unreported Number of Novel Coronavirus (2019-nCoV) Cases in China in the First Half of January 2020: A Data-Driven Modelling Analysis of the Early Outbreak. J Clin Med. 2020;9.

11. Nishiura H, Kobayashi T, Miyama T, Suzuki A, Jung S, Hayashi K, et al. Estimation of the asymptomatic ratio of novel coronavirus infections (COVID-19). Int J Infect Dis. 2020;94:154–5. doi:10.1016/j.ijid.2020.03.020.

12. Liu Z, Chu R, Gong L, Su B, Wu J. The assessment of transmission efficiency and latent infection period in asymptomatic carriers of SARS-CoV-2 infection. Int J Infect Dis. 2020;99:325–7.

13. Hou C, Chen J, Zhou Y, Hua L, Yuan J, He S, et al. The effectiveness of quarantine of Wuhan city against the Corona Virus Disease 2019 (COVID-19): A well-mixed SEIR model analysis. Journal of Medical Virology. 2020;92:841–8. doi:10.1002/jmv.25827.
